# Unmasking the Great Mimic: An Atypical Presentation of Giant Cell Arteritis With Recurrent and Isolated Diplopia

**DOI:** 10.7759/cureus.94445

**Published:** 2025-10-13

**Authors:** George Pandarakalam Thomas, Joyita Barua, Ahmet Ubur, Paul Bolaji

**Affiliations:** 1 Internal Medicine, Dorset County Hospital, Dorchester, GBR; 2 Neurosurgery, University Hospital of Wales, Cardiff, GBR; 3 Stroke Medicine, Dorset County Hospital, Dorchester, GBR

**Keywords:** binocular diplopia, c-reactive protein, erythrocyte sedimentation rate, giant cell arteritis, intima-media thickness, ophthalmic artery, temporal artery ultrasound, transient ischemic attack, vasculitis

## Abstract

Giant cell arteritis (GCA) is a well-recognized vasculitis, typically presenting with headaches, scalp tenderness, jaw claudication, polymyalgia rheumatica, or constitutional symptoms. In stroke and transient ischemic attack (TIA) clinics, GCA is primarily considered in cases of visual loss due to ophthalmic artery involvement. However, diplopia as an isolated manifestation of GCA is exceedingly rare, making early recognition challenging. We present the case of a 75-year-old man who attended our TIA clinic with seven recurrent episodes of transient binocular diplopia. Notably, he lacked the classical features of GCA, including headache, scalp tenderness, jaw claudication, or visual loss. There was no history of fever, night sweats, weight loss, or other systemic symptoms. His neurological examination was unremarkable, and he denied any recent trauma or medication changes. However, routine inflammatory markers revealed a significantly elevated erythrocyte sedimentation rate (ESR) and CRP, raising suspicion for an underlying inflammatory process. An urgent rheumatology assessment and temporal artery ultrasound demonstrated increased intima-media thickness in the superficial temporal, frontal, and axillary arteries, confirming the diagnosis of GCA. He was promptly initiated on high-dose prednisolone (1 mg/kg) with a structured 16.5-month tapering regimen. Following treatment, his diplopia resolved completely, and inflammatory markers normalized. At both three-month and six-month follow-ups, he remained asymptomatic. To our knowledge, this is an extremely rare case of GCA presenting with isolated, recurrent diplopia rather than the typical pattern of visual loss. We propose that the underlying mechanism may involve intracranial vasculitis affecting the posterior circulation, particularly the midbrain and pons, or ischemia of the extraocular muscles due to ophthalmic artery involvement. This case highlights the protean nature of GCA, reinforcing its reputation as the “great mimic.” Clinicians should maintain a high index of suspicion for GCA in older patients presenting with transient diplopia, even in the absence of classical symptoms. Routine ESR and CRP testing in TIA clinics could facilitate early diagnosis, preventing irreversible visual or cerebrovascular complications.

## Introduction

Giant cell arteritis (GCA) is a chronic, inflammatory condition primarily affecting medium and large arteries, characterized by granulomatous inflammation and infiltration of arterial walls, which can lead to luminal narrowing and ischemic complications, including permanent visual loss if untreated [1]. It is a critical medical emergency in ophthalmology due to its potential to cause preventable visual loss if diagnosed and treated promptly. GCA is the most common vasculitis in individuals aged 50 years and older, and it does not typically occur in those under 50 years of age. It has a higher prevalence in females, with a female-to-male ratio ranging from 1.4:1 to 2.9:1 [1]. Common symptoms include severe physical and mental fatigue, proximal upper extremity muscle pain, headaches, scalp tenderness, visual loss, and jaw claudication. However, diagnosis is often delayed for several months due to the gradual or insidious onset of symptoms, which can complicate timely intervention [2].

Two forms of GCA are recognized: a cranial form involving the medium-caliber temporal artery, causing temporal arteritis, and an extracranial form affecting large vessels, primarily the thoracic aorta and its branches, including the vertebrobasilar system supplying the midbrain and pons [2]. The variability in affected arteries leads to atypical presentations in some patients, lacking classical features such as headache or temporal artery tenderness [3]. Previous case reports have documented rare presentations, such as transient diplopia without classical symptoms, highlighting the need for heightened clinical suspicion to prevent severe outcomes like permanent visual loss or stroke [4,5]. If untreated, GCA can progress to irreversible ischemic complications, underscoring the urgency of early diagnosis and management.

Standard treatment involves high-dose corticosteroids, typically prednisolone at 1 mg/kg, to rapidly control inflammation and prevent progression to visual loss or other ischemic events [6]. More recently, IL-6 inhibitors, such as tocilizumab, have been introduced as steroid-sparing agents to reduce corticosteroid-related adverse effects and improve long-term outcomes [7]. This case report aims to elucidate a rare clinical presentation of GCA to enhance clinicians’ understanding and support the development of definitive management guidelines.

This article was previously presented as a meeting abstract at the BAPIO YDF Conference in Bristol on May 10, 2025.

## Case presentation

A 75-year-old man presented to the emergency department following seven episodes of transient binocular diplopia over one month, each lasting approximately 10-15 minutes. He described seeing images side by side but denied headaches, scalp tenderness, jaw or tongue claudication, visual loss, polymyalgia rheumatica symptoms, or other ischemic symptoms suggestive of a transient ischemic attack (TIA) or stroke. He reported no fever, night sweats, weight loss, recent trauma, new medication use, palpitations, sweating, or diarrhea. He denied ptosis, and the diplopia was not time-dependent. The patient had no significant past medical history, including no history of migraines (with or without aura), atrial fibrillation, antiphospholipid antibody syndrome (APS), systemic lupus erythematosus (SLE), or hematologic disorders such as thrombocytosis or polycythemia. He was not on any regular medications and had no known allergies.

On examination, visual field tests and pursuit eye movements were normal, with no nystagmus or diplopia noted during eye movements. Motor, sensory, coordination, and cranial nerve examinations were unremarkable, and no pathological reflexes were elicited. Auscultation of the heart and major blood vessels, as well as the ECG, yielded normal findings.

Laboratory tests revealed elevated inflammatory markers, as shown in Table 1, including a CRP of 77 mg/L and an erythrocyte sedimentation rate (ESR) of 58 mm/h. Platelet count and WBC count were within normal limits. MRI and CT of the head showed no acute hemorrhage or stroke but noted an incidental arachnoid cyst in the left temporal region. Based on these findings, TIA or infection was deemed unlikely, and GCA was suspected, prompting urgent referral to the ophthalmology and rheumatology departments. Key differentials considered included migrainous auras or ophthalmoplegic/acephalgic brainstem migraines (which can cause transient diplopia without headache), embolic events from intermittent atrial fibrillation or thrombotic disorders (e.g., APS or SLE), and other vasculitides. Similar previous reports have described transient diplopia as a rare initial presentation of GCA due to ischemic involvement of cranial nerves or brainstem vessels, often resolving with treatment but risking progression to visual loss [8,9]. GCA’s pathology involves granulomatous inflammation of arterial walls, leading to occlusion and ischemia; if untreated, it can result in permanent blindness or stroke, emphasizing the need for prompt recognition.

**Table 1:** Blood investigation results on presentation and after treatment ESR, erythrocyte sedimentation rate

Investigation	Reference range	On presentation	After one month of treatment
Hemoglobin	130-170 g/L	150 g/L	123 g/L
White cell count	4-10 × 10⁹/L	7 × 10⁹/L	7.9 × 10⁹/L
Platelets	150-410 × 10⁹/L	182 × 10⁹/L	383 × 10⁹/L
ESR	1-30 mm/h	58 mm/h	1 mm/h
CRP	0-5 mg/L	77 mg/L	1 mg/L

**Figure 1:**
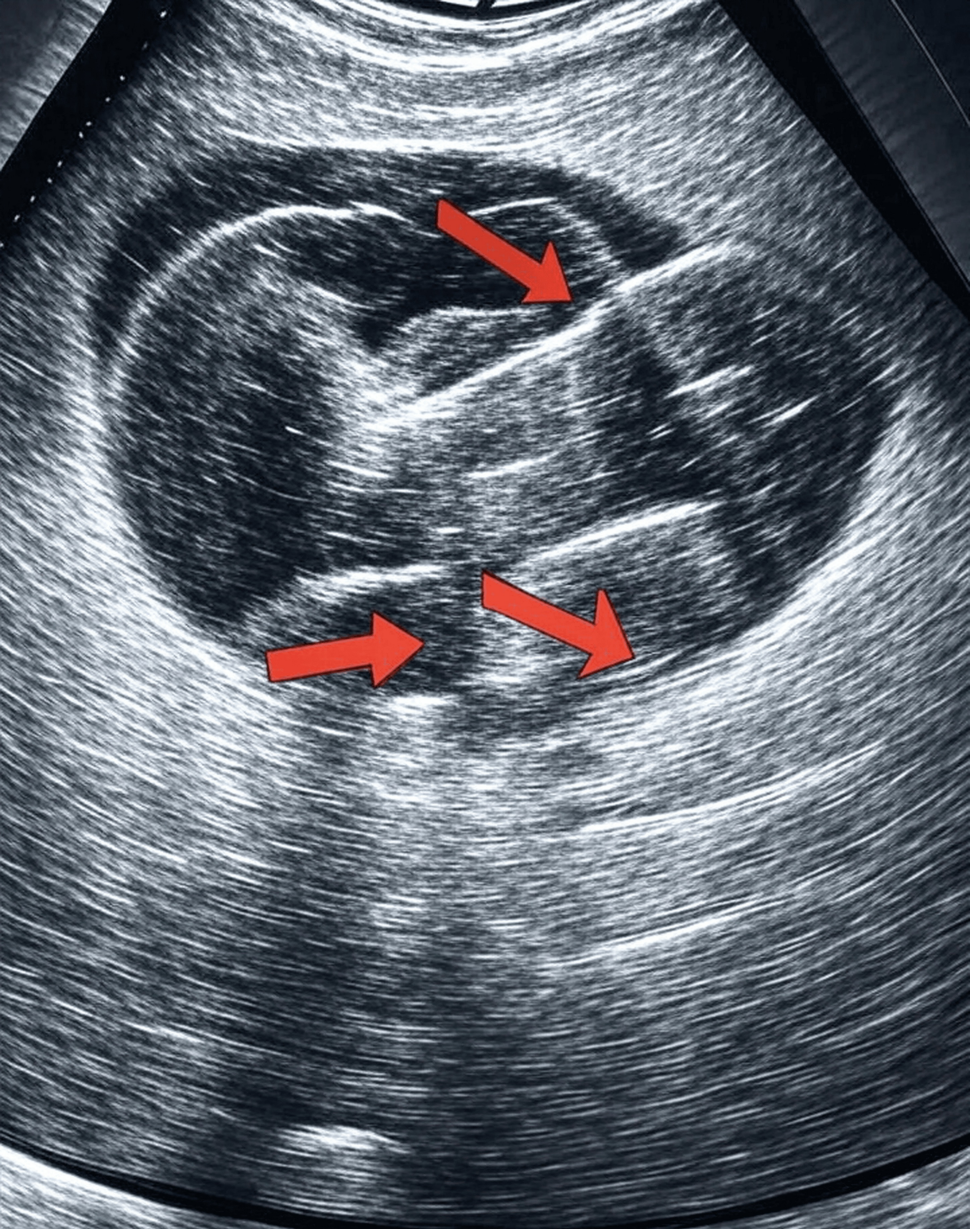
Temporal artery ultrasound showing increased intima-media thickness in the superficial temporal artery The red arrows indicate areas of thickened intima media, consistent with GCA. GCA, giant cell arteritis

The rheumatology department performed an urgent temporal artery ultrasound the following day. The ultrasound findings are detailed in Figure 1, showing increased intima-media thickness and reduced compressibility in the superficial temporal, frontal, and axillary arteries of both sides, corresponding to the “halo sign” (a hypoechoic rim around the artery lumen indicative of inflammation) and the “compressibility sign” (reduced arterial compressibility due to wall thickening). These findings confirm the diagnosis of GCA. No significant stenosis was observed in the ultrasound, and as the patient did not undergo a temporal artery biopsy, no data on skipped lesions are unfortunately available. The patient was started on high-dose prednisolone (80 mg, 1 mg/kg) with a planned 16.5-month tapering regimen. A CT of the chest, abdomen, and pelvis and a CT aortogram showed no involvement of other major vessels.

The ophthalmology team found no evidence of relative afferent pupillary defect. Fundoscopy revealed normal optic nerve discs bilaterally and normal visual fields. A follow-up ophthalmology review one month later showed no significant ocular findings.

## Discussion

This case report describes a patient with recurrent transient diplopia diagnosed with GCA based on positive ultrasound findings, as detailed in Figure 1. Diplopia may result from intraocular or neuromuscular causes, cerebrovascular accidents, or cranial nerve palsies [4]. Ischemia of the extraocular muscles or their blood supply may contribute to diplopia [5]. The ultrasound findings, showing bilateral increased intima-media thickness and reduced compressibility in the superficial temporal, frontal, and axillary arteries, correspond to the “halo sign” (a hypoechoic rim around the artery lumen indicative of inflammatory edema) and the “compressibility sign” (reduced arterial wall elasticity due to thickening), suggesting ischemia-driven arterial wall narrowing due to granulomatous inflammation, a hallmark of GCA. Clinical examinations and investigations are critical for diagnosis and treatment in such cases, with diplopia serving as a warning sign to investigate vascular causes of ocular manifestations.

Age is a significant factor in older adults with transient ocular symptoms, particularly when risk factors like hypertension or diabetes suggest ischemic causes [6]. This patient had a history of hypertension, which heightened suspicion for GCA as a differential diagnosis. Tamhankar et al. emphasized the importance of thorough history-taking to identify GCA symptoms such as jaw pain or headache, yet this patient lacked these features, underscoring the challenge of atypical presentations [6]. His referral to the stroke clinic for suspected TIA highlights how GCA’s atypical presentations can be mistaken for other conditions.

Historically, CRP and ESR have been key inflammatory markers in GCA diagnosis, with CRP being more sensitive and the combination of both offering high specificity [7,8]. Hayreh et al. noted a significant association between these markers and jaw claudication, which was absent in this case [7]. Additionally, Oh et al. reported thrombocytosis in GCA, though this patient’s platelet count was normal [9]. The elevated CRP (77 mg/L) and ESR (58 mm/h), as shown in Table 1, supported the GCA diagnosis despite the absence of classical symptoms, suggesting a need for further studies on the relationship between inflammatory markers and diplopia.

Temporal artery biopsy is the gold standard for GCA diagnosis but is now less favored due to complications such as insufficient sample size and invasiveness [10]. Ultrasound has become the preferred initial diagnostic modality, with Moiseev et al. advocating that clinical suspicion combined with positive ultrasound findings is sufficient for diagnosis [11]. The ultrasound findings in Figure 1 confirmed arterial wall thickening, enabling early diagnosis and treatment, while a CT aortogram showed no involvement of other major vessels. A digital subtraction angiogram could be considered to further assess vascular patency but was not pursued given the diagnostic clarity from ultrasound and the patient’s stable condition.

Permanent or transient diplopia is a rare GCA presentation and may precede visual loss [12]. Similar reports have documented transient diplopia as an early ischemic manifestation of GCA, often resolving with treatment but with a risk of progression to visual loss if untreated [8,9,12]. Early corticosteroid treatment has been shown to resolve ocular symptoms and prevent progression to visual loss [5,13]. The patient reported no adverse effects from the corticosteroid treatment, and therefore, no such effects were included in the report. This patient’s diplopia resolved following high-dose prednisolone (1 mg/kg) with a 16.5-month tapering regimen, with no recurrence at three- and six-month follow-ups. Differentials for diplopia in this case included migrainous auras or ophthalmoplegic/acephalgic brainstem migraines, embolic events from atrial fibrillation or thrombotic disorders (e.g., APS or SLE), cerebrovascular accidents, cranial nerve palsies, and other vasculitides, all of which were ruled out through clinical and blood investigations and imaging findings. Continued monitoring will provide insights into the long-term effects of treatment.

## Conclusions

This case underscores the importance of recognizing transient diplopia as a rare presentation of GCA, particularly in the absence of classical symptoms. This case’s uniformity lies in its consistency with documented atypical presentations and diagnostic approaches for GCA, while its rarity highlights the importance of considering GCA in patients with transient diplopia. The conclusions are supported by the data, including the patient’s resolution of symptoms following treatment, normalization of elevated inflammatory markers after therapy, and ultrasound findings consistent with bilateral arterial wall changes. Early diagnosis and treatment with corticosteroids can prevent irreversible complications and promote recovery. Clinicians should consider GCA as a differential diagnosis in older adults with systemic or ocular symptoms, supported by routine inflammatory marker testing and ultrasound imaging.

## References

[1] Bas-Lando M, Breuer GS, Berkun Y, Mates M, Sonnenblick M, Nesher G (2007). The incidence of giant cell arteritis in Jerusalem over a 25-year period: annual and seasonal fluctuations. Clin Exp Rheumatol.

[2] Paroli M, Caccavale R, Accapezzato D (2024). Giant cell arteritis: advances in understanding pathogenesis and implications for clinical practice. Cells.

[3] Winkler A, True D (2018). Giant cell arteritis: 2018 review. Mo Med.

[4] Ross AG, Jivraj I, Rodriguez G (2019). Retrospective, multicenter comparison of the clinical presentation of patients presenting with diplopia from giant cell arteritis vs other causes. J Neuroophthalmol.

[5] Nealon C, Canania R (2022). Diplopia as the presenting symptom in giant cell arteritis. Can J Optom.

[6] Tamhankar MA, Biousse V, Ying GS (2013). Isolated third, fourth, and sixth cranial nerve palsies from presumed microvascular versus other causes: a prospective study. Ophthalmology.

[7] Hayreh SS, Podhajsky PA, Raman R, Zimmerman B (1997). Giant cell arteritis: validity and reliability of various diagnostic criteria. Am J Ophthalmol.

[8] Parikh M, Miller NR, Lee AG (2006). Prevalence of a normal C-reactive protein with an elevated erythrocyte sedimentation rate in biopsy-proven giant cell arteritis. Ophthalmology.

[9] Oh LJ, Wong E, Andrici J, McCluskey P, Smith JE, Gill AJ (2018). Full blood count as an ancillary test to support the diagnosis of giant cell arteritis. Intern Med J.

[10] Bowling K, Rait J, Atkinson J, Srinivas G (2017). Temporal artery biopsy in the diagnosis of giant cell arteritis: does the end justify the means?. Ann Med Surg (Lond).

[11] Moiseev SV, Smitienko I, Bulanov N, Novikov PI (2019). The role of temporal artery biopsy in patients with giant-cell arteritis is debated. Ann Rheum Dis.

[12] Haering M, Holbro A, Todorova MG (2014). Incidence and prognostic implications of diplopia in patients with giant cell arteritis. J Rheumatol.

[13] Chazal T, Clavel G, Leturcq T, Philibert M, Lecler A, Vignal-Clermont C (2024). Characteristics and prognosis of binocular diplopia in patients with giant cell arteritis. J Neuroophthalmol.

